# Intra- and Interobserver Variability in Magnetic Resonance Imaging Measurements in Rectal Cancer Patients

**DOI:** 10.3390/cancers13205120

**Published:** 2021-10-13

**Authors:** Peter Grimm, Martina Kastrup Loft, Claus Dam, Malene Roland Vils Pedersen, Signe Timm, Søren Rafael Rafaelsen

**Affiliations:** 1 Department of Radiology, Vejle Hospital, University Hospital of Southern Denmark, 7100 Vejle, Denmark; martina.kastrup.loft@rsyd.dk (M.K.L.); claus.dam@rsyd.dk (C.D.); Malene.Roland.Vils.Pedersen@rsyd.dk (M.R.V.P.); Soeren.Rafael.Rafaelsen@rsyd.dk (S.R.R.); 2 Department of Regional Health Research, University of Southern Denmark, 5230 Odense, Denmark; signe.timm@rsyd.dk; 3 Research Unit, Kolding Hospital, University Hospital of Southern Denmark, 6000 Kolding, Denmark

**Keywords:** rectal cancer, lymph node staging, intraobserver variability, interobserver variability, radiology

## Abstract

**Simple Summary:**

Colorectal cancer is the second most common cancer and was the second most common cause of cancer-related death in Europe in 2018. Accurate lymph node staging in primary rectal cancer staging is essential for the selection of the proper treatment regimen. In 2018, The European Society of Gastrointestinal and Abdominal Radiology published consensus recommendations for primary rectal cancer staging, and suggested that lymph nodes be assessed by size, morphology, and location in- or outside the mesorectal fascia. Our study aimed to assess the inter- and intraobserver variability in size, apparent diffusion coefficient measurements, and morphological characterization among inexperienced and experienced radiologists. Our data indicate that subjective variables like morphological characteristics are less reproducible than numerical variables, regardless of the level of experience of the observers.

**Abstract:**

Colorectal cancer is the second most common cancer in Europe, and accurate lymph node staging in rectal cancer patients is essential for the selection of their treatment. MRI lymph node staging is complex, and few studies have been published regarding its reproducibility. This study assesses the inter- and intraobserver variability in lymph node size, apparent diffusion coefficient (ADC) measurements, and morphological characterization among inexperienced and experienced radiologists. Four radiologists with different levels of experience in MRI rectal cancer staging analyzed 36 MRI scans of 36 patients with rectal adenocarcinoma. Inter- and intraobserver variation was calculated using interclass correlation coefficients and Cohens-kappa statistics, respectively. Inter- and intraobserver agreement for the length and width measurements was good to excellent, and for that of ADC it was fair to good. Interobserver agreement for the assessment of irregular border was moderate, heterogeneous signal was fair, round shape was fair to moderate, and extramesorectal lymph node location was moderate to almost perfect. Intraobserver agreement for the assessment of irregular border was fair to substantial, heterogeneous signal was fair to moderate, round shape was fair to moderate, and extramesorectal lymph node location was substantial to almost perfect. Our data indicate that subjective variables such as morphological characteristics are less reproducible than numerical variables, regardless of the level of experience of the observers.

## 1. Introduction

Colorectal cancer is the second most common cancer, with 500,000 new cases in Europe in 2018, and was the second most common cause of cancer-related death with 243,000 deaths in Europe in the same year [1]. Rectal cancer accounts for 27–58% of all colorectal cancer cases [2]. Accurate lymph node (LN) staging in rectal cancer patients is essential for the selection of the proper treatment regimen. Hence, LN involvement is an independent prognostic factor predicting overall survival and local recurrence [3]. Historically, LNs were assessed using size criteria alone. Brown et al. concluded that the prediction of LN involvement in rectal cancer with magnetic resonance imaging (MRI) is improved by using morphologic characteristics instead of size criteria [4].

In contrast, Gröne et al. [5] found no improvement in the accuracy of LN staging by using morphological criteria. The European Society of Gastrointestinal and Abdominal Radiology (ESGAR) published consensus recommendations for the primary staging and restaging of rectal cancer using MRI. The assessment of size, morphology, and mesorectal/extramesorectal LN involvement forms the cornerstone of LN staging [6]. Brown et al. showed substantial interobserver agreement by dividing LN into “involved” and “non-involved” groups with a kappa value (κ) of 0.71 among radiologists with 5 to 10 years of experience in MRI [4]. 

It is unclear whether the recommended assessment criteria for LN staging presented by ESGAR are reproducible among radiologists with different experience levels. Although apparent diffusion coefficient (ADC) measurement has no crucial role in primary rectal staging, recent studies suggest that the ADC measurement could improve the diagnostic accuracy of LN staging [7,8,9]. To our knowledge, no inter- and intraobserver variability study of lymph node size, ADC, and morphological characteristics has been published.

Therefore, this study aims to assess the inter- and intraobserver variability of size, morphology, mesorectal/extramesorectal LN involvement, and potentially beneficial LN characteristics (e.g., ADC measurements) among inexperienced and experienced radiologists.

## 2. Materials and Methods

### 2.1. Patients and MRI

A total of 155 patients with rectal cancer from the Department of Surgery, Vejle Hospital, Denmark underwent an MRI of the rectum between 1 January and 31 December 2018, and were considered for inclusion in this intra- and interobserver variation study of lymph node staging in locally advanced rectal cancer. Inclusion criteria consisted of (1) biopsy-proven rectal adenocarcinoma and (2) locally advanced disease with positive LN stage defined by primary T2-weighted magnetic resonance imaging (T2W-MRI) and diffusion-weighted magnetic resonance imaging (DWI-MRI) conducted either on a 1.5 Tesla or 3 Tesla MRI scanner (Philips Medical Systems, Best, the Netherlands) using the same scanning protocol. 

After localizer scans, fast T2-weighted (T2W) spin-echo sequences were obtained. The scans, which included 3 mm axial slices at a 90° angle to the tumor axis, were prepared by the MRI radiographer assisted by a radiologist to ensure perpendicular images. No contrast enhancement was used. DWI was performed perpendicular to the tumor using an echo-planar imaging (EPI) factor of 61. Five different b values (strength and timing of the gradients to generate DWI) were used by applying diffusion-sensitive gradients: b  =  0, b  =  200, b  =  400, b  =  600, and b  =  800 s/mm^2^. The first series was a set of image sequences formed by echo-planar spin-echo T2W imaging (b  =  0). The next series formed gradients in the x, y, and z directions and formed isotropic images which were obtained by calculating diffusion vector projections of the three directions. ADC maps of the isotropic images were created automatically by the Philips Ingenia software. Patients were scanned in the supine position. Bowel cleansing was not performed, and no oral or rectal contrast media were administered. A total of 36 patients were eligible and included in the study. The remaining 119 patients were excluded due to negative lymph node stage, previous surgical intervention, benign tumor, or relapse of primary cancer.

The study was conducted according to the guidelines of the Declaration of Helsinki and approved by the Institutional Review Board (May 2020) of the University Hospital of Southern Denmark (Journal number: 20/25129) and the local Danish Data Protection Agency. An additional informed consent was not required in the present retrospective study since no additional diagnostic information was generated from the 36 primary MRI staging scans from 2018.

### 2.2. Lesion Selection 

One to four lymph nodes per patient were selected at random by SRR (observer 4). A total of 104 lymph nodes were marked with image numbers and arrowheads and were plotted into a blinded lymph node assessment form. To minimize observer bias, the observer who selected the lymph nodes read the cases more than four weeks after selection.

### 2.3. Lymph Node Assessment

All observer characteristics are presented in Table 1. A three-monitor workstation setup was used with one allocated radiological information system (RIS) (Carestream Health, Inc.), with an 18.5” display (Lenovo, China) for patient selection, and two allocated picture archiving and communication systems (PACSs) (Medical Insight, Valby, Denmark) with displays (21.3" Monitor CCL358i2 from: Totoku, JVCENWOOD Corporation, Kanagawa, Japan) for picture evaluation. Each observer assessed the lymph nodes independently and reported on a pre-printed lymph node assessment form with one patient ID and one to four lymph nodes in random order. The selected MRI images were input into a separate research file within the RIS. No additional clinical information beyond the presence of histopathologically proven rectum cancer was provided. There were three numerical variables: length and width in millimeters, apparent diffusion coefficient (ADC) in mm^2^/s, and four binary non-numerical variables (i.e., round shape, irregular border, heterogeneous signal, and extramesorectal lymph node). Observers were encouraged to use electronic calipers for measurements. T2W-MRI images with 3 mm slice thickness were used to measure length and width and assess the binary non-numerical variables. The ADC map was used for ADC measurements. A second read of the MRI images was performed three months after the first read, and the MRI images were presented in random order.

### 2.4. Statistical Analysis

Intra- and interobserver agreement of the numerical variables was estimated by the interclass correlation coefficient (ICC). A two-way random-effects absolute agreement model was used to estimate the interobserver ICC and corresponding 95% confidence intervals (CIs) of the first and second reads. A similar two-way random-effects absolute agreement model was used for intraobserver agreement to estimate the ICC and corresponding 95% CI for each observer. All models were performed separately for length, width, and ADC measurements. ICC values were interpreted using the following cut-offs: below 0.50, poor; between 0.50 and 0.75, fair; between 0.75 and 0.90, good; above 0.90, excellent [10].

Bland–Altman plots were produced for length, width, and ADC measurements, plotting the mean of the two reads against the difference and limits of agreement.

Intraobserver agreement of the non-numerical variables from the first and second reads was calculated using Cohens-kappa (κ). The observer (observer 3) with the highest kappa value (irregular border: 0.69; heterogeneous signal: 0.53; round shape: 0.56; extramesorectal lymph node: 0.95) was used as the “gold standard” when estimating pairwise interobserver agreement of the non-numerical variables at the first and second reads. All calculations were performed separately for irregular border, heterogeneous signal, round shape, and extramesorectal lymph node.

Kappa values were interpreted using the following cut-offs: <0 poor, 0.00–0.20 slight, 0.21–0.40 fair, 0.41–0.60 moderate, 0.61–0.80 substantial, 0.81–1 almost perfect [11]. A 95% CI was estimated using bootstrapping.

Statistical analysis was performed using STATA 16 ( Stata Corp., LLC 4905 Lakeway Drive, College Station, TX, USA).

## 3. Results

### 3.1. Numerical Variables

Interobserver agreement of the length measurements for the first read was excellent, and it was good for the second read, with ICCs of 0.94 for both reads. The intraobserver agreement was excellent, with ICCs of 0.94–0.98. For width measurements, the interobserver agreement of the first read was excellent, with an ICC of 0.93, and good for the second read (0.89). The intraobserver agreement was excellent for observers 2 and 3 with ICCs ranging 0.95–0.96, and for observers 1 and 4 the agreement was good, with ICCs ranging 0.89–0.92. For ADC measurements, the interobserver ICCs for the first and second reads were fair, ranging 0.73–0.79. The intraobserver agreement was good for observers 1, 2, and 3, with ICCs ranging 0.84–0.89; it was fair for observer 4, with an ICC of 0.79 (Table 2).

**Figure 1:**
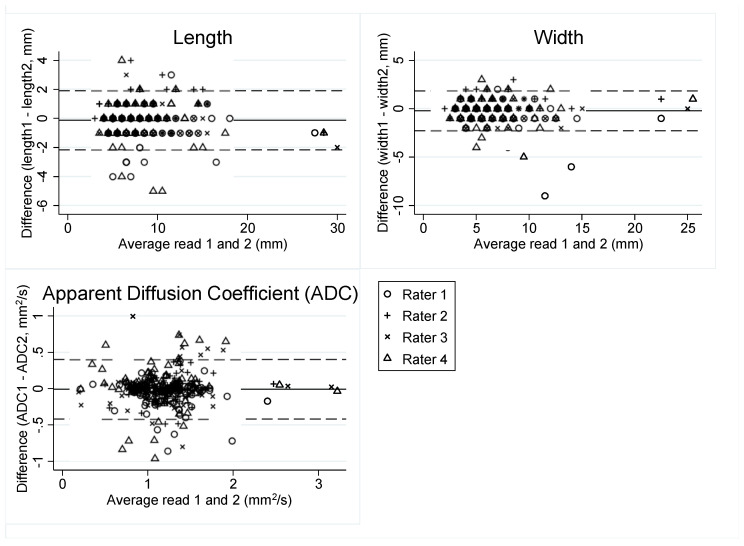
Bland–Altman plots showing intraobserver agreement as average in read 1 and 2 (x-axis), plotted against the difference between read 1 and 2 (y-axis). The solid horizontal line corresponds to the mean difference (length: −0.13 mm, width: −0.22 mm, ADC: −0.01 mm^2^/s).

Intraobserver agreement on the numerical variables showed narrow limits of agreement with the mean towards zero in the Bland–Altman plots (Figure 1).

### 3.2. Morphological and Location Characteristics

**Figure 2:**
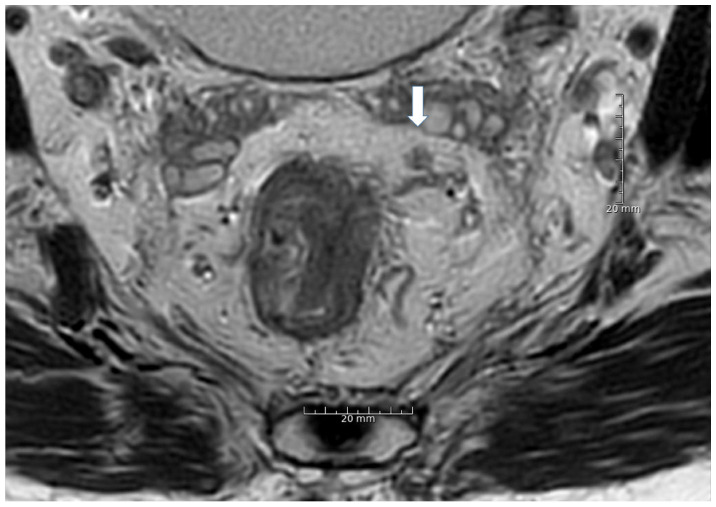
Arrow: A 6 mm heterogeneous lymph node with irregular border located at 2 o’clock within the mesorectal fat.

**Figure 3:**
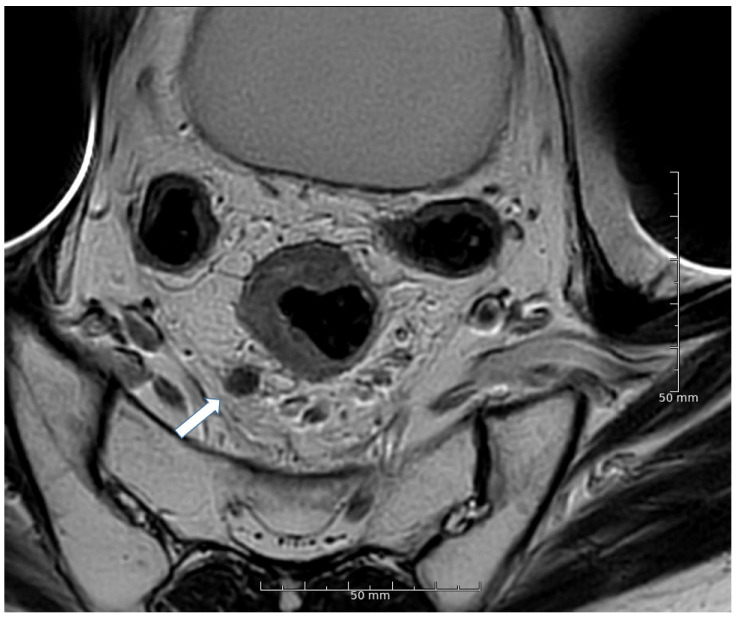
Arrow: A rounded 9 mm mesorectal lymph node with irregular border at 7 o’clock.

**Figure 4:**
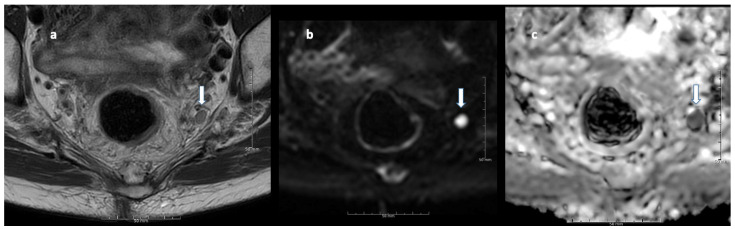
A 10 mm large extramesorectal lymph node (lateral LN) at the left internal iliac indicated with arrows at T2W image (**a**), diffusion weighted MRI b800 (**b**), and with low signal on the apparent diffusion coefficient (ADC) map indicating diffusion restriction (**c**).

Table 3 shows kappa values (κ) for inter-and intraobserver variability in assessing irregular border, heterogeneous signal, round shape, and extramesorectal LN location. Figure 2, Figure 3 and Figure 4 show MRI images of characteristic LNs. Interobserver agreement of irregular border assessment was fair to substantial, the heterogeneous signal assessment was slight to moderate, and the round shape assessment was slight to substantial. Extra mesorectal LN location assessments were fair to almost perfect. Intraobserver agreement of irregular border assessment ranged from fair to almost perfect, heterogeneous signal assessment was fair to substantial, round shape assessment was fair to substantial, and agreement in lateral/extramesorectal LN location ranged from moderate to almost perfect. 

## 4. Discussion

The current study shows that the assessment of LN size and ADC measurements with MRI was highly reproducible, regardless of the level of observer experience, in patients with positive LNs. On the other hand, the morphological assessment of LNs with MRI showed lower reproducibility, except for extramesorectal LN location with high observer agreement.

The preoperative identification of patients with LN-negative disease with a good prognosis is important. It helps to select the patients who are likely to have a better outcome with surgical intervention alone [3]. The workshop training of radiologists could possibly improve the LN assessment by avoiding the classification of small nodes with an oval, homogeneous, and regular boundary as LN-positive disease and thereby resulting in less preoperative overtreatment.

An MRI of the pelvic region is the standard for rectal cancer staging. ESGAR’s consensus recommendations on the assessment of LN involvement in primary rectal cancer staging by MRI fundamentally depends on size assessment, morphology, and location characteristics [6,12].

Kono et al. [13] published a node-for-node comparative study of specimen and histology and found rectal cancer metastases in LN as small as 1 mm in diameter. They concluded that size criteria alone cannot accurately predict the LN involvement of rectal cancer.

Previous studies have shown mixed results regarding the benefits of using morphological criteria in addition to size. Kim et al. [14] performed a single-observer study with one experienced radiologist. They concluded that the presence of individual criteria like spiculated or indistinct border, or heterogenic signal of the LNs, in addition to LN size could be helpful to predict LN involvement. Brown et al. [4] performed an interobserver study with two experienced radiologists who had at least five years of experience in the MRI staging of rectal cancer. They showed that the predictive value of LN size criteria alone was poor because of substantial overlap between benign and malignant LNs. However, diagnostic accuracy improved through the use of morphological criteria such as the assessment of border and signal intensity. Furthermore, they showed good reproducibility by categorizing LNs into “involved” and “non-involved” groups, with LNs regarded as positive if either an irregular border or a mixed-signal intensity was present. Gröne et al. [5] conducted a retrospective single-observer study in which one radiologist with over 20 years of experience in the MRI staging of rectal cancer analyzed previously obtained MRI images of patients with histopathologically verified rectum cancer. They found no significant improvement in the diagnostic accuracy between size criteria alone and a combination of size and morphological criteria.

All inter- and intraobserver agreements within numerical variables were reasonably high, with good to excellent agreement for length and width measurements and fair to good agreements for ADC; all CIs were overlapping, indicating no significant difference in measurements obtained by inexperienced and experienced observers. Our high reproducibility of ADC is in accordance with the findings of Kwee et al. [9]. However, no unambiguous results have been published showing whether ADC measurements can be used for LN staging in rectal cancer patients. Heijnen et al. [8] showed that DWI-MRI and ADC measurements could improve LN visualization in primary rectal cancer staging, but the diagnostic accuracy was not improved. Nevertheless, interobserver agreement was excellent for ADC measurements. Lambregts et al. [7] found that ADC measurements on MRI scans in patients with rectal cancer after chemoradiation may improve LN characterization. However, this can be difficult in small LNs, as 28% of malignant LNs in rectal cancer are less than 3 mm in size [15].

Our data regarding LN location within or without the mesorectal fascia show reasonably high reproducibility among all observers. In correlation with the study of Kim et al. [16], which showed extramesorectal LN involvement to be a major factor for locoregional recurrence, our data indicate a good intra- and interobserver agreement for the presence of extramesorectal LNs. This is important since the lateral extramesorectal LNs are not removed by standard total mesorectal excision (TME), and even neoadjuvant chemoradiotherapy with TME is not sufficient to prevent lateral local recurrence in enlarged nodes [17,18]. Some consider lateral nodal disease to represent metastatic disease that is not amenable to treatment, and persistently enlarged lateral nodes after chemoradiotherapy indicate a high risk of local recurrence [19]. Lateral lymph node dissection may improve locoregional control in patients with low rectal cancer and abnormal lateral LNs, but larger studies are warranted [20].

MRI LN staging in rectal cancer patients remains complex, and more research in the field is needed to improve the diagnostic accuracy. A recent study by Ding et al. suggested that artificial intelligence (AI) might add diagnostic accuracy in the evaluation of metastatic LNs in patients with rectal cancer [21]. Other methods are also being tested, and there is a need for further improvement in the LN staging of rectal cancer [22,23,24,25,26,27,28,29,30,31]. 

Colorectal cancers with deficient mismatch repair (dMMR) display a greater inflammatory response and local infiltration of lymphocytes in tumor and peritumoral tissue; dMMR can arise during DNA replication. The distinct clinicopathological features of dMMR colorectal cancer affect the accuracy of preoperative N staging [32,33]. Exactly how this affects the interobserver variation remains unclear.

A limitation of our study is the absence of an absolute reference for the morphological characteristics of the LNs. All MRI scans assessed were of patients with histopathologically verified rectal adenocarcinoma. Since no histopathological node-by-node LN stage was correlated with our findings, pairwise kappa values were calculated for interobserver agreement using the most consistent observer (observer 3). This approach seemed satisfactory for the purpose of interobserver variation for morphological criteria in LN assessment. However, it did not provide any clinical value for the interpretation of whether an LN is benign or malignant. The prospective node-by-node approach used by Brown et al. [4] would have been preferable.

Although our patient population of 36 patients was relatively small and the LNs were not correlated directly with histopathology, we found that the assessment of 104 LNs contributed valuable data regarding the reproducibility of the traditionally used LN assessment characteristics of length, width, and morphology, as well as newer, potentially beneficial factors like ADC among radiologists of different experience levels.

All observers assessed the LNs on similar PACS stations to minimize variances in image quality. Since the use of electronic magnification was neither recommended nor prohibited at the beginning of this study, no data regarding the use of electronic magnification was recorded. The use of electronic magnification might have affected the measurement accuracy, predominantly on the small lymph nodes.

## 5. Conclusions

Our data indicate that MRI numerical LN variables are reproducible regardless of the level of experience of the observers, whereas subjective variables like morphological characteristics are less reproducible.

Prospective node-by-node validation studies are warranted to investigate whether quantitative ADC measurements can improve LN staging by imaging in rectal cancer.

## Figures and Tables

**Table 1:** Observer characteristics.

Observer	Experience MRI Rectal Staging	Title
Observer 1	0 years	Resident
Observer 2	0 years	Resident
Observer 3	10 years	Consultant
Observer 4	>20 years	Consultant

All observers were employed at the Department of Radiology, Vejle Hospital, Denmark.

**Table 2:** Inter- and intraobserver interclass correlation coefficient (ICC) and lymph node (LN) measurements.

	Length		Width		ADC	
Interobserver	No. of LN *	ICC (95% CI)	No. of LN *	ICC (95% CI)	No. of LN *	ICC (95% CI)
1st Read	98	0.94 (0.92–0.96)	99	0.93 (0.91–0.95)	89	0.79 (0.72–0.85)
2nd Read	100	0.94 (0.87 0.97)	100	0.89 (0.82–0.93)	96	0.73 (0.65–0.80)
**Intraobserver**	No. of LN *	**ICC (95% CI)**	No. of LN *	**ICC (95% CI)**	No. of LN *	**ICC (95% CI)**
Observer 1	103	0.95 (0.90–0.97)	104	0.89 (0.80–0.93)	95	0.84 (0.72–0.90)
Observer 2	102	0.96 (0.94–0.98)	102	0.95 (0.90–0.98)	95	0.89 (0.84–0.90)
Observer 3	102	0.98 (0.97–0.99)	102	0.96 (0.91–0.98)	99	0.85 (0.79–0.90)
Observer 4	99	0.94 (0.91–0.96)	99	0.92 (0.88–0.95)	93	0.79 (0.69–0.85)

ADC, apparent diffusion coefficient; No., number; LN, lymph node; ICC, interclass correlation coefficient; CI, confidence interval; * statistical analysis was performed on complete datasets. The number of LNs does vary because of failure to report on the LN assessment form.

**Table 3:** Kappa values for lymph node measurements.

	Irregular Border		Heterogeneous Signal		Round Shape		Extra Mesorectal LN	
Interobserver	LNs *	Kappa (95% CI) (Agreement)	LNs *	Kappa (95% CI) (Agreement)	LNs *	Kappa (95% CI) (Agreement)	LNs *	Kappa (95% CI) (Agreement)
Observer 1								
1st Read	103	0.48 (0.31–0.65) (74%)	102	0.30 (0.12–0.48) (65%)	103	0.28 (0.08–0.48) (72%)	102	0.68 (0.40–0.96) (95%)
2nd Read	104	0.44 (0.28–0.59) (71%)	104	0.21 (0.04–0.39) (61%)	104	0.53 (0.39–0.68) (76%)	104	0.74 (0.50–0.97) (95%)
Observer 2								
1st Read	101	0.42 (0.25–0.60) (71%)	101	0.30 (0.11–0.49) (70%)	101	0.26 (0.15–0.37) (55%)	99	0.94 (0.79–1.08) (99%)
2nd Read	104	0.54 (0.39–0.69) (78%)	104	0.38 (0.21–0.56) (69%)	104	0.53 (0.38–0.68) (76%)	104	0.75 (0.56–0.95) (94%)
Observer 4								
1st Read	100	0.44 (0.26–0.62) (72%)	100	0.21 (0.02–0.40) (62%)	101	0.21 (0.02–0.40) (64%)	97	0.56 (0.29–0.82) (91%)
2nd Read	102	0.51 (0.34–0.67) (75%)	102	0.27 (0.09–0.45) (64%)	102	0.50 (0.33–0.67) (76%)	102	0.95 (0.84–1.06) (99%)
**Intraobserver**	LNs *	**Kappa (95% CI) (Agreement)**	LNs *	**Kappa (95% CI) (Agreement)**	LNs *	**Kappa (95% CI) (Agreement)**	LNs *	**Kappa (95% CI) (Agreement)**
Observer 1	104	0.50 (0.34–0.66) (76%)	104	0.39 (0.22–0.56) (69%)	104	0.38 (0.26–0.51) (66%)	104	0.81 (0.58–1.04) (97%)
Observer 2	102	0.40 (0.24–0.56) (71%)	102	0.23 (0.04–0.41) (67%)	102	0.56 (0.39–0.72) (79%)	101	0.68 (0.47–0.90) (93%)
Observer 3	103	0.69 (0.54–0.83) (84%)	102	0.53 (0.38–0.69) (76%)	103	0.56 (0.38–0.73) (80%)	102	0.95 (0.83–1.06) (99%)
Observer 4	99	0.58 (0.42–0.74) (79%)	100	0.40 (0.22–0.59) (71%)	100	0.46 (0.28–0.64) (75%)	97	0.65 (0.42–0.89) (93%)

LNs, lymph nodes; * statistical analysis was performed on complete datasets. The number of LNs varies because of the failure to report on the LN assessment form.

## Data Availability

Data sharing is not applicable to this article.
